# Effects of medium‐ and long‐chain fatty acids on acetaminophen‐ or rifampicin‐induced hepatocellular injury

**DOI:** 10.1002/fsn3.1641

**Published:** 2020-05-19

**Authors:** Jun Yang, Ting Peng, Jiyong Huang, Guohua Zhang, Jiaheng Xia, Maomao Ma, Danwen Deng, Deming Gong, Zheling Zeng

**Affiliations:** ^1^ State Key Laboratory of Food Science and Technology Nanchang University Nanchang China; ^2^ Jiangxi Province Key Laboratory of Edible and Medicinal Plant Resources Nanchang University Nanchang China; ^3^ College of Food and Technology Nanchang University Nanchang China; ^4^ School of Environmental and Chemical Engineering Nanchang University Nanchang China; ^5^ Department of Biomedicine New Zealand Institute of Natural Medicine Research Auckland New Zealand

**Keywords:** apoptosis, drug‐induced liver injury, inflammatory cytokines, long‐chain fatty acids, medium‐chain fatty acids, oxidative stress

## Abstract

Drug‐induced liver injury (DILI) is one of the common adverse effects of drug therapy, which is closely associated with oxidative stress, apoptosis, and inflammation response. Medium‐chain fatty acids (MCFA) were reported to relieve inflammation and attenuate oxidative stress. However, little has been known about the hepatoprotective effects of MCFA in DILI. In the present study, acetaminophen (AP) and rifampicin (RFP) were used to establish DILI models in LO2 cells, and the cytoprotective effects of MCFA on hepatocellular injury were investigated. Results showed that the optimal condition for the DILI model was treatment with 10 mM AP or 600 µM RFP for 24 hr. LCFA treatment markedly reduced the cell viability and increased the activities of alanine aminotransferase, aspartate aminotransferase, and lactate dehydrogenase. Meanwhile, LCFA treatment aggravated cell apoptosis, mitochondrial dysfunction, and oxidative stress. The mRNA and protein expression levels of inflammatory cytokines (IL‐1β and TNF‐α) were significantly elevated by LCFA. In contrast, MCFA treatment did not significantly affect cell viability, apoptosis, oxidative, stress and inflammation, and it did not produce the detrimental effects on DILI models. Therefore, we proposed that MCFA may be more safe and suitable than LCFA as nutrition support or the selection of daily dietary oil and fat for the patients with DILI.

## Graphical abstract

In this study, we investigated the optimal conditions for establishing the DILI model and the cytoprotective effects of MCFA on the model. Our results found that MCFA decreased the percentages of apoptotic cells, mitochondrial membrane potential as well as attenuated oxidative stress compared with LCFA. MCFA also down‐regulated the mRNA and protein expression of inflammatory cytokines.

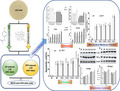

## 1 INTRODUCTION

The liver is a vital organ of mammals and primarily responsible not only for maintaining nutrient homeostasis by regulating protein, carbohydrate, and fat metabolism, but also exerting detoxification by decomposing and transforming various drugs and toxins, and excreting certain metabolites in the body. Therefore, when some drugs were taken overdose, they may overwhelm the liver self‐detoxification capacity, leading to liver injury (Bernal, Lee, Wendon, Larsen, & Williams, 2015). Drug‐induced liver injury (DILI) is one of the frequent side effects of drug therapy, which can cause a broad spectrum of liver disorders, including steatohepatitis, cholestasis, cirrhosis, hepatocarcinoma, and even death (Björnsson & Hoofnagle, 2016; Zuquan, Kejian, Haibo, & Qiang, 2015). A retrospective study in China found an estimated annual incidence of 23.80 cases per 100,000 persons, which was higher than in western countries (Shen et al., 2019). The annual incidence of DILI was estimated as 2.7 per 100,000 adults, and the major cause was overdose of acetaminophen (AP) used in the United States (Vega et al., 2017). In Asian countries, antituberculosis drugs and herbal medicines were the most common causative drugs in DILI (Sobhonslidsuk, Poovorawan, Soonthornworasiri, Pan‐Ngum, & Phaosawasdi, 2016). AP, commonly used as an analgesic and antipyretic drug, may easily induce DILI when it was used overdose (Ramachandran & Jaeschke, 2019). Rifampicin (RFP), known as the first‐line antituberculosis drugs, caused DILI when given separately or given in combination with others (Benson et al., 2016). Therefore, AP or RFP (always combined with isoniazid) was a classical drug for establishing the DILI model in vivo or in vitro. In this study, we first determined the conditions of AP and RFP to establish a DILI model in LO2 cells and then investigated the effects of fatty acid (FA) on the hepatocellular injury.

Fatty acid with 8–12 carbon atoms are regarded as medium‐chain fatty acids (MCFA) including octanoic acid (C8:0), decanoic acid (C10:0), and lauric acid (C12:0). MCFA are mainly present in human milk fat (∼6.93%) and several plant oils, including coconut oil (∼58.4%), palm kernel oil (∼64.4%), and *Cinnamomum camphora* seed kernel oil (∼91.6%) (Duan, Shin, Qin, Kwon, & Lee, 2019; Jing et al., 2015; Macaire et al., 2013; Sivakanthan, Jayasooriya, & Madhujith, 2019). FA with more than 12 carbon atoms are considered to be long‐chain fatty acids (LCFA), which are found mostly as components of the triacylglycerols of edible oils and fats. Compared with LCFA, MCFA are absorbed easily and supply energy rapidly. In addition to the faster energy metabolism property mentioned above, MCFA has been investigated for its hepatoprotective effects. Several studies showed that a diet rich in MCFA improved lipid metabolism and lipid accumulation in rats with nonalcoholic fatty liver disease by regulating apoptosis, oxidative stress, and inflammatory responses (Juárez‐Hernández, Chávez‐Tapia, Uribe, & Barbero‐Becerra, 2015). Furthermore, another study found that MCFA attenuated lipopolysaccharide‐induced liver injury through down‐regulating necroptotic and inflammatory signaling pathways (Zhang et al., 2018). However, these studies were hardly focusing on the relationship between FA and DILI, and many patients with DILI consumed LCFA (especially oleic acid) in daily oil and fat diets due to lack of scientific references. In this study, we found that LCFA aggravated the hepatocellular injury. Therefore, this study may contribute to the development of functional oils and provide knowledge on the selection of oil and fat for patients with DILI.

## 2 MATERIALS AND METHODS

### 2.1 Materials

LO2 cells (normal human liver cell line HL7702) were obtained from the Shanghai Institute of Biochemistry and Cell Biology, Chinese Academy of Sciences. RPMI‐1640 medium was purchased from Hyclone Laboratories Inc. AP, RFP, and fetal bovine serum were from Sigma‐Aldrich Co. Sodium caprylate, sodium caprate, sodium laurate, and sodium oleate were from Aladdin Industrial Corp. Dimethyl sulfoxide (DMSO), MTT dye, and apoptosis assay kits were from Solarbio. Aspartate aminotransferase (AST), alanine aminotransferase (ALT), lactate dehydrogenase (LDH), and all antioxidant capacity assay kits were purchased from Nanjing Jiancheng Bioengineering Institute. The primary antibodies (anti‐IL‐1β, anti‐TNF‐α, and anti‐β‐actin) were from Abcam, and secondary antibodies horseradish peroxidase (HRP‐conjugated goat anti‐rabbit/mouse immunoglobulin G) were from Servicebio. All other chemicals were of analytical grade.

### 2.2 Cellular model of drug‐induced liver injury

LO2 cells were grown in RPIM 1640 supplemented with 10% fetal bovine serum and maintained in a humidified CO_2_‐regulated incubator with a 5% CO_2_ atmosphere at 37°C. The AP stock solution was dissolved in PBS to 200 mM, and the RFP stock solution was dissolved in DMSO to 1,000 µM and diluted by the serum‐free medium. LO2 cells were seeded into 96‐well plates at a density of 1 × 10^4^ cells per well and grew overnight at 37°C in 5% CO_2_. Then, the cells were treated with AP at 5–20 mM or RFP at 200–800 µM. Control cells were treated with vehicle alone. Plates were incubated at 37°C for 24, 48, and 72 hr; then, the cell viability was determined using MTT assay. In another set of experiments, cells were seeded into 24‐well plates at a density of 5 × 10^4^ cells per well. After treated as above, plates were incubated at 37°C for 24 hr. Then, the media were collected to measure the activities of AST and ALT using activity determination kits, according to the manufacturer's instructions. Then, the apoptotic cells were measured by double staining with Hoechst 33342/PI assay. Briefly, the treated cells were washed three times with cold PBS and stained with Hoechst 33342/PI at 4°C for 20 min in the dark, then washed twice with cold PBS and observed under a fluorescence microscope. AST and ALT assay, and apoptosis observation were conducted as confirmation experiments to support the MTT assay.

### 2.3 Measurement of cell viability

Fatty acid (including octanoic acid, decanoic acid, lauric acid, and oleic acid) were dissolved in PBS to form the stock solution at 8 mM and diluted by the serum‐free medium. For the viability assay, LO2 cells were seeded into 96‐well plates at a density of 1 × 10^4^ cells per well and cultured overnight. Then, the cells were treated with different concentrations of FA (0, 50, 100, 200, 400, and 800 µM) for 24 hr. For further studies, LO2 cells were also treated with 20 mM AP and 600 µM RFP for 24 hr and then incubated with 200 µM FA for an additional 24 hr. Then, 10 µl of MTT solution was added to each well and incubated for 4 hr at 37°C. Next, the supernatant was carefully removed, the purple formazan crystals were dissolved in 150 µl DMSO, and the absorbance value at 490 nm was measured by a SpectraMax^®^ absorbance reader (Molecular Devices).

### 2.4 Lactate dehydrogenase (LDH) assay

Lactate dehydrogenase assay was conducted as a confirmation experiment to support the MTT assay. The level of LDH released from injured or dead cells was measured as an indicator of cytotoxicity. The LO2 cells were seeded at 1 × 10^4^ cells/well onto a 96‐well plate for 12 hr and treated as the procedure for MTT assay. The supernatant was collected to measure the activity of LDH using an activity determination kit, according to the manufacturer's instructions. The absorbance at 450 nm was then determined by a SpectraMax^®^ absorbance reader.

### 2.5 Apoptosis assay

LO2 cells were seeded at a density of 1 × 10^6^ cells per well onto 6‐well plates and cultured overnight. Then, the wells were divided into the following groups: The cells were treated with 20 mM AP or 600 µM RFP for 24 hr and then incubated with 200 µM octanoic acid (C8:0 group), decanoic acid (C10:0 group), lauric acid (C12:0 group), and oleic acid (C18:1 group) for an additional 24 hr, the cells treated with 20 mM AP or 600 µM RFP for 24 hr and then incubated with serum‐free medium containing PBS or 0.1% DMSO for an additional 24 hr (naturally restoring group, NR group), and the cells treated with serum‐free medium (normal control group, NC group). After the treated cells were rinsed three times with cold PBS and harvested by trypsinization, they were washed twice with cold PBS followed by centrifugation at 112 *g* for 10 min. Then, the cell pellet was resuspended in 500 µl binding buffer followed by incubation with Annexin V‐FITC (5 µl) and PI (5 µl) for 15 min at room temperature in the dark. The excitation wavelength was 488 nm, and the emission wavelength was 525 nm. A flow cytometer with BD Accuri C6 software (FCM, Becton‐Dickinson) was used to determine the apoptosis rate after acquiring 10,000 cells. Furthermore, apoptotic hepatocytes were identified by morphology, after the treated cells were washed three times with cold PBS and stained with Hoechst 33342/PI at 4°C for 20 min in the dark, then washed twice with cold PBS and observed under a fluorescence microscope.

### 2.6 Measurement of mitochondrial membrane potential (MMP)

The treated cells were evaluated for the change in MMP using Rhodamine 123 (Rh‐123) cationic dye and a flow cytometer. Briefly, the cells were harvested and resuspended in complete RPMI 1640 media (with 10% FBS) containing Rh‐123 (5 µg/ml) and kept at 37°C for 30 min. Next, the cells were washed twice with PBS, followed by centrifugation at 447 *g* for 5 min to remove the unreacted Rh‐123 dye. Finally, the cell pellet was resuspended in 500 µl PBS and detected by flow cytometry. Ten thousand cells were acquired and analyzed by BD Accuri C6 software.

### 2.7 Determination of intracellular antioxidant indexes

Intracellular antioxidant indexes, including total antioxidant capacity (T‐AOC), levels of malondialdehyde (MDA), and glutathione (GSH), and activities of superoxide dismutase (SOD) and catalase (CAT) were determined with assay kits according to the instructions of the manufacturers. Briefly, the cells were harvested and incubated in a chilled cell lysis buffer for 30 min. The cells were centrifuged at 16,099 *g* for 10 min at 4°C, and the supernatant was collected to measure antioxidant indexes. Total protein content in the supernatant was determined by BCA protein assay kit (Beyotime Biotechnology), using bovine serum albumin as a standard. All assays were conducted at least in six replicates, and values given are average of these replicates.

### 2.8 Quantitative real‐time polymerase chain reaction (q‐PCR)

The expression levels of the IL‐1β, IL‐6, IL‐8, MCP‐1, and TNF‐α mRNAs were analyzed by q‐PCR. The total RNA was isolated from a cell sample using TRIzol Reagent (CWBIO) according to the manufacturer's protocol. Reverse transcription was conducted using a Quantscript RT Kit (Tiangen Biotech Co). PCR was carried out in a total volume of 10 µl using an SYBR Premix Kit (Takara Bio Inc.) on a 7900 HT Fast Real‐Time PCR System (Applied Biosystems) with the following program: 95°C for 30 s, followed by 40 cycles of 5 s at 95°C and 30 s at 60°C. The primers (Table 1) were designed using Primer Express and synthesized by Sangon Biotech. GADPH was used as a housekeeping gene to normalize the expression levels of the target genes. The relative expression ratio of the mRNA was calculated by the comparative critical threshold method. Data are expressed as a fold change relative to the NC group.

**TABLE 1:** The sequences of primers used for quantitative real‐time PCR analysis

Cytokines	Forward (5′−3′)	Reverse (5′−3′)
IL‐1β	ATGATGGCTTATTACAGTGGCAA	GTCGGAGATTCGTAGCTGGA
IL‐6	CCTGAACCTTCCAAAGATGGC	TTCACCAGGCAAGTCTCCTCA
IL‐8	ACTGAGAGTGATTGAGAGTGGAC	AACCCTCTGCACCCAGTTTTC
MCP‐1	CAGCCAGATGCAATCAATGCC	TGGAATCCTGAACCCACTTCT
TNF‐α	CCTCTCTCTAATCAGCCCTCTG	GAGGACCTGGGAGTAGATGAG
GAPDH	CAATGACCCCTTCATTGACC	GACAAGCTTCCCGTTCTCAG

### 2.9 Western blot analyses

The cells were lysed in RIPA buffer (Beyotime Biotechnology) with a protease inhibitor PMSF (Beyotime Biotechnology) and centrifuged at 16,099 *g* for 10 min at 4°C to obtain the supernatant. Cellular total protein concentration was quantified by BCA protein assay kit according to the manufacturer's protocol. Equal amounts of protein (20 µg per lane) were separated by 12% SDS‐PAGE and transferred onto PVDF membranes (0.22 µm, Millipore). After blocking with Tris‐buffered saline Tween‐20 (TBST) containing 5% skimmed milk powder for 2 hr at room temperature, the membranes were incubated with different primary antibodies: anti‐IL‐1β (1:4,000, mouse monoclonal antibody), anti‐TNF‐α (1:1,500, mouse monoclonal antibody), and anti‐β‐actin (1:2,000, rabbit polyclonal antibody) at 4°C overnight. Then, the cells were incubated with secondary HRP‐conjugated goat anti‐rabbit/mouse IgG (1:3,000) for 2 hr at room temperature after washed by TBST three times. At last, the protein bands on the membranes were visualized with the enhanced ECL detection system (Bio‐Rad) after washed by TBST three times. Protein bands were quantitatively analyzed using AlphaEaseFC software and normalized to β‐actin.

### 2.10 Statistical analysis

Statistical Package for the Social Sciences 19.0 software (SPSS, Inc.) was used to process all data. Data were presented as the mean ± standard deviation (*SD*). The differences between groups were analyzed by one‐way analysis of variance (ANOVA), followed by the independent *t* test. Values at *p* < .05 and *p* < .01 were considered to be statistically significant and very significant.

## 3 RESULTS

### 3.1 Cell viabilities, activities of ALT and AST, and apoptosis in AP‐ or RFP‐treated LO2 cells

**FIGURE 1:**
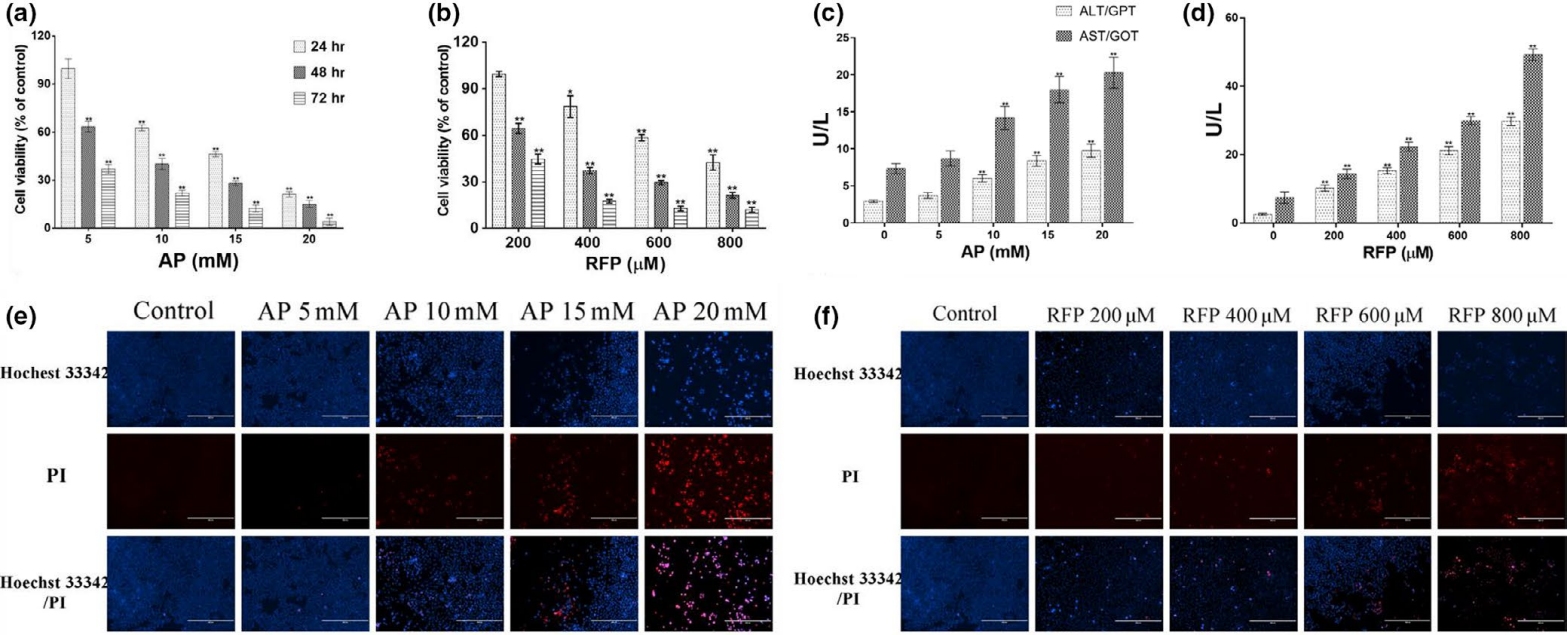
Cytotoxicity of AP and RFP on LO2 cells. The proliferation activities of LO2 cells treated with different concentrations of AP (a, 5, 10, 15, and 20 mM) and RFP (b, 200, 400, 600, and 800 μM) for 24, 48, and 72 hr. The activities of ALT and AST in the supernatant in LO2 cells treated with different concentrations of AP (c, 0, 5, 10, 15, and 20 mM) and RFP (d, 0, 200, 400, 600, and 800 μM) for 24 hr. Apoptotic cells were observed under a fluorescence microscope with Hoechst 33342/PI costaining assay, after treatment with AP (e, 0–20 mM) and RFP (f, 0–800 μM) for 24 hr. Data represent the means ± *SD* (*n* = 6). *p < .05, **p < .01 compared with the NC group

LO2 cells were used to establish a hepatocyte injury model by AP or RFP treatment. To determine the optimal damage conditions, LO2 cells were treated with different concentrations of AP or RFP for 24, 48, and 72 hr, and the cell viability was determined by MTT assay. As shown in Figure 1a,b, with the increases in the concentration of AP or RFP and treatment time, the survival rates of LO2 cells decreased gradually in a dose‐ and time‐dependent manner. Cell viability was significantly decreased in the AP‐treated group from 10 to 20 mM for 24 hr and the RFP‐treated group from 400 to 800 µM for 24 hr compared to the control group (*p* < .01). The results also showed that the half‐maximal inhibitory concentrations (IC_50_) of AP and RFP in LO2 cells treated for 24, 48, and 72 hr were 13.15, 7.439, and 3.543 mM and 696.5, 298.2, and 164.1 µM, respectively.

To further evaluate the hepatic injury effects of AP or RFP on LO2 cells for 24 hr, ALT and AST assay and Hoechst 33342/PI assay were conducted. The results showed that compared to the control group, the activities of both ALT and AST significantly increased in a concentration‐dependent manner when the concentrations of AP and RFP were higher than 5 mM and 200 µM, respectively (*p* < .01, Figure 1c,d). Additionally, the results from fluorescence microscopy showed that treatments with 10–20 mM of AP or 400–800 µM of RFP evoked a marked increase in the number of apoptotic cells in drug‐induced hepatocellular injury, whereas treatment with 5 mM of AP or 200 µM of RFP remained unchanged or increased slightly compared with the control group (Figure 1e,f). These results showed that LO2 cells can be used as an in vitro model for drug‐induced hepatotoxicity, and the optimal concentration on the cells was 10 mM of AP or 600 µM of RFP for 24 hr.

### 3.2 Effects of FA on the cell viability and toxicity of LO2 cells and AP‐ or RFP‐treated LO2 cells

**FIGURE 2:**
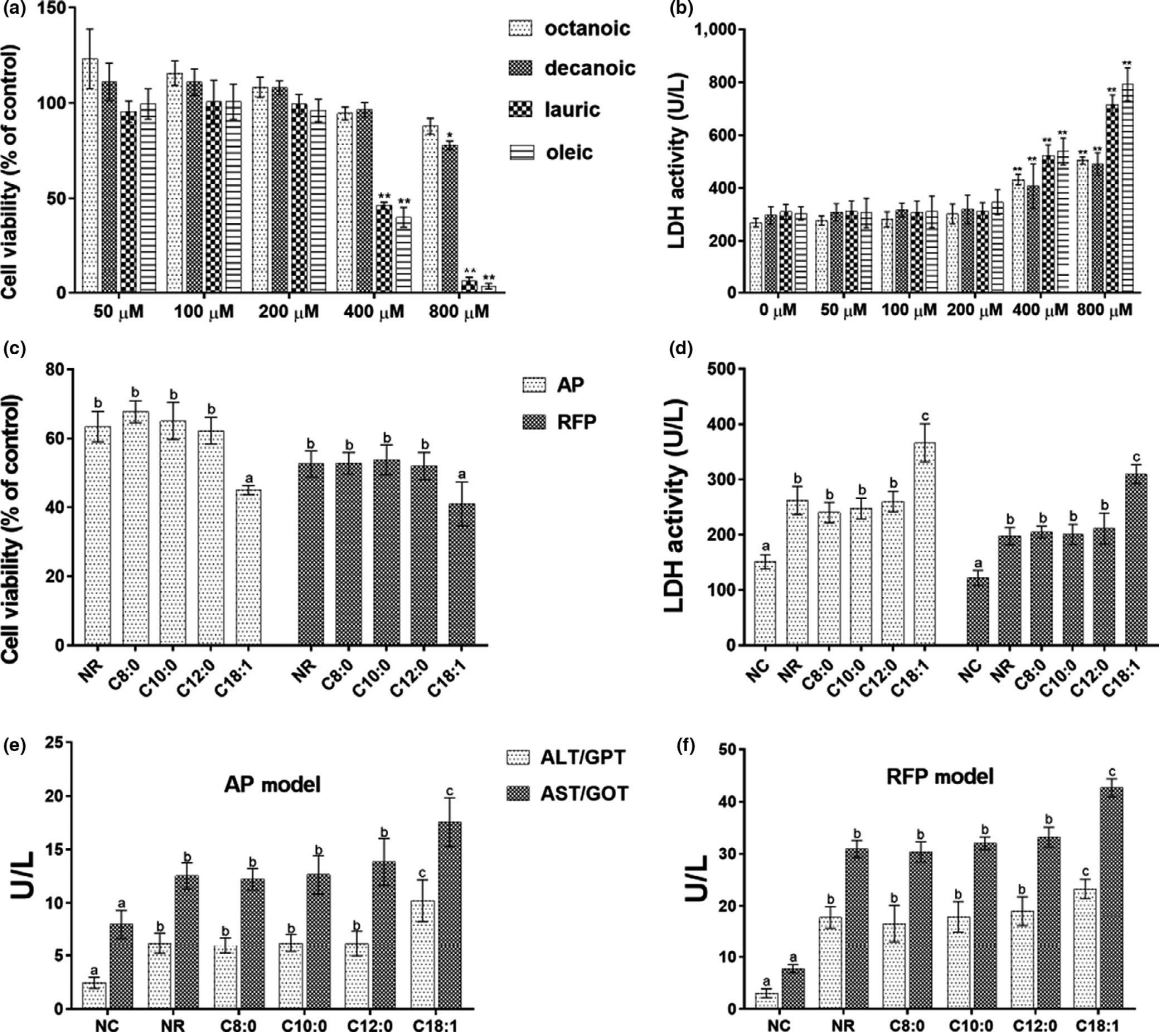
Effect of FA on the cell viability and toxicity in LO2 cells and AP‐ or RFP‐induced LO2 cell model. The proliferation activities of LO2 cells (a) and LDH activity in cell supernatant (b) after 24‐hr exposure to FA at different concentrations. Data represent the means ± *SD* (*n* = 6). *p < .05, **p < .01 compared with the NC group. The proliferation activities of AP‐ or RFP‐induced LO2 cell model (c) and LDH activity in the supernatant (d) after 24‐hr exposure to FA at different concentrations. The levels of ALT and AST in the supernatant in AP‐induced LO2 cell model (e) and RFP‐induced LO2 cell model (f) after treated with 200 μM FA for 24 hr. Mean values (*n* = 6) with the same letter are not significantly different (*p* > .05)

The MTT and LDH assay were performed to determine the effect of FA on cytotoxicity of LO2 cells and hepatoprotection of AP‐ or RFP‐damaged cell model (AP or RFP model). As shown in Figure 2a, the proliferation of LO2 cells was reduced when the concentrations of FA were higher than 200 µM. Especially, lauric acid and oleic acid at concentrations higher than 200 µM induced significant cytotoxic responses compared with the control group (*p* < .01). In addition, AP or RFP model treated with 200 µM of MCFA showed cell viability similar to the NR group, whereas treatment with 200 µM of oleic acid showed a significant decrease in the cell viability (*p* < .01, compared with the NR group) (Figure 2c). Meanwhile, as shown in Figure 2b,d, the LDH activity was dramatically increased when the FA concentration exceeded 200 µM, and there were no significant differences in LDH activity between MCFA and NR group in AP or RFP model. However, the AP or RFP model treated with 200 µM of oleic acid showed a significant increase in LDH activity (*p* < .01, compared with the NR group). To further evaluate the effects of FA on AP or RFP model, ALT and AST assay were conducted (Figure 2e,f). Results showed that the activities of ALT and AST in C18:1 group were significantly increased compared with MCFA groups (*p* < .01). These results showed that less than 200 µM FA did not cause obvious cytotoxicity. Compared with the NR group, treatment with 200 µM LCFA worsen liver cell death but treatment with 200 µM MCFA did not aggravate cell injury in AP or RFP model.

### 3.3 Effects of MCFA and LCFA on apoptosis in AP‐ or RFP‐treated LO2 cells

**FIGURE 3:**
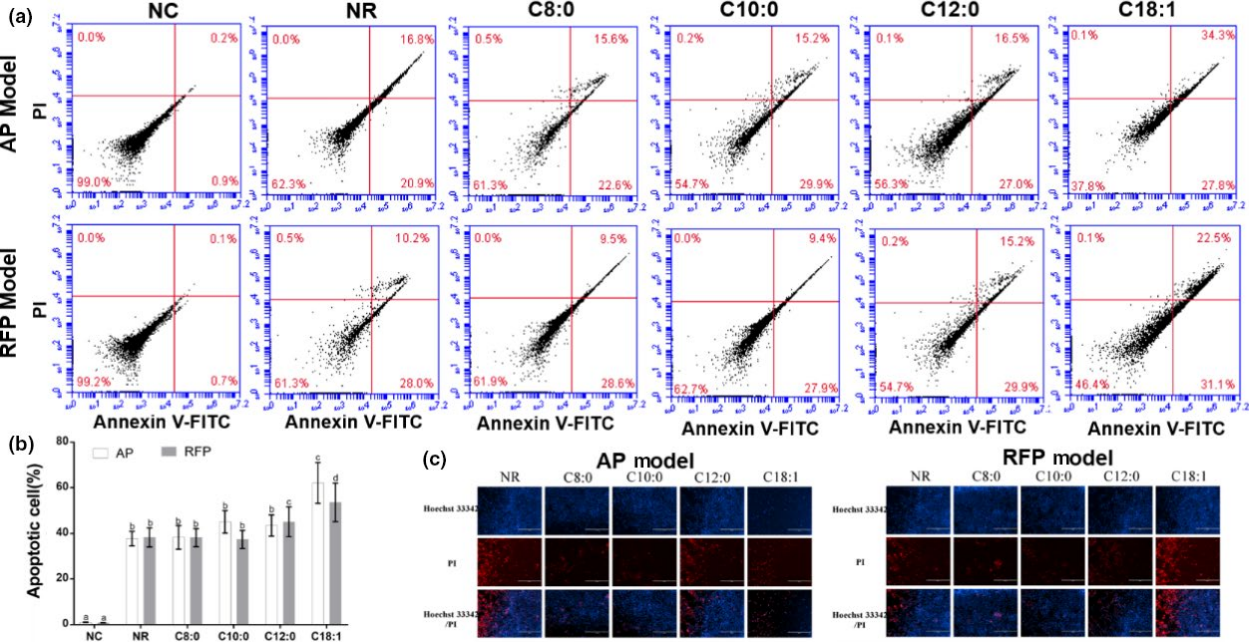
Effect of 200 μM FA on the apoptosis in AP‐ and RFP‐induced LO2 cells model. The cells were incubated with FA for 24 hr. Apoptotic cells were quantified by FCM (a) stained with Annexin V‐FITC/PI, and the apoptotic proportion was determined by FCM (b). Mean values (*n* = 3) with the same letter are not significantly different (*p* > .05). The apoptotic cells of AP model (c) and RFP (d) were stained with Hoechst 33342/PI and then observed by a fluorescence microscope. Numbers within quadrants represent the percentages of cells in early and median apoptosis (Annexin V + PI −; lower right) and in late apoptosis and necrosis (Annexin V + PI +; upper right)

In order to determine whether MCFA and LCFA may influence apoptosis in AP‐ or RFP‐induced liver injury cell model, double staining with Hoechst 33342/PI assay was used to identify morphological features of apoptotic cells and double staining with Annexin V‐FITC/PI was used to determine apoptosis rate. As shown in Figure 3a,b, compared with the LCFA group, the percentage of late apoptotic and necrotic cells in MCFA groups were significantly decreased (from 34.3% to 15.6%, 15.2%, and 16.5% in AP model and from 22.5% to 9.5%, 9.4%, and 15.2% in RFP model, respectively), and the percentages of apoptotic cells in the NR and the MCFA groups were also significantly decreased (from 62.1% to 37.7%, 38.2%, 45.1%, and 43.5% in AP model and from 53.6% to 38.2%, 37.1%, 37.3%, and 45.1% in RFP model, respectively). Moreover, the results also indicated that the apoptotic ratios in the MCFA groups were not significantly different from the NR group, except that C12:0 group in RFP model. As shown in Figure 3c,d, apoptotic cells identified by morphology also verified the results of the apoptotic ratio by flow cytometry. The results showed that C18:1 treatment evoked a marked increase in the number of apoptotic cells, whereas it remained unchanged or slightly declined in MCFA groups compared with the NR group.

### 3.4 Effect of MCFA and LCFA on mitochondrial membrane potential in AP‐ or RFP‐treated LO2 cells

**FIGURE 4:**
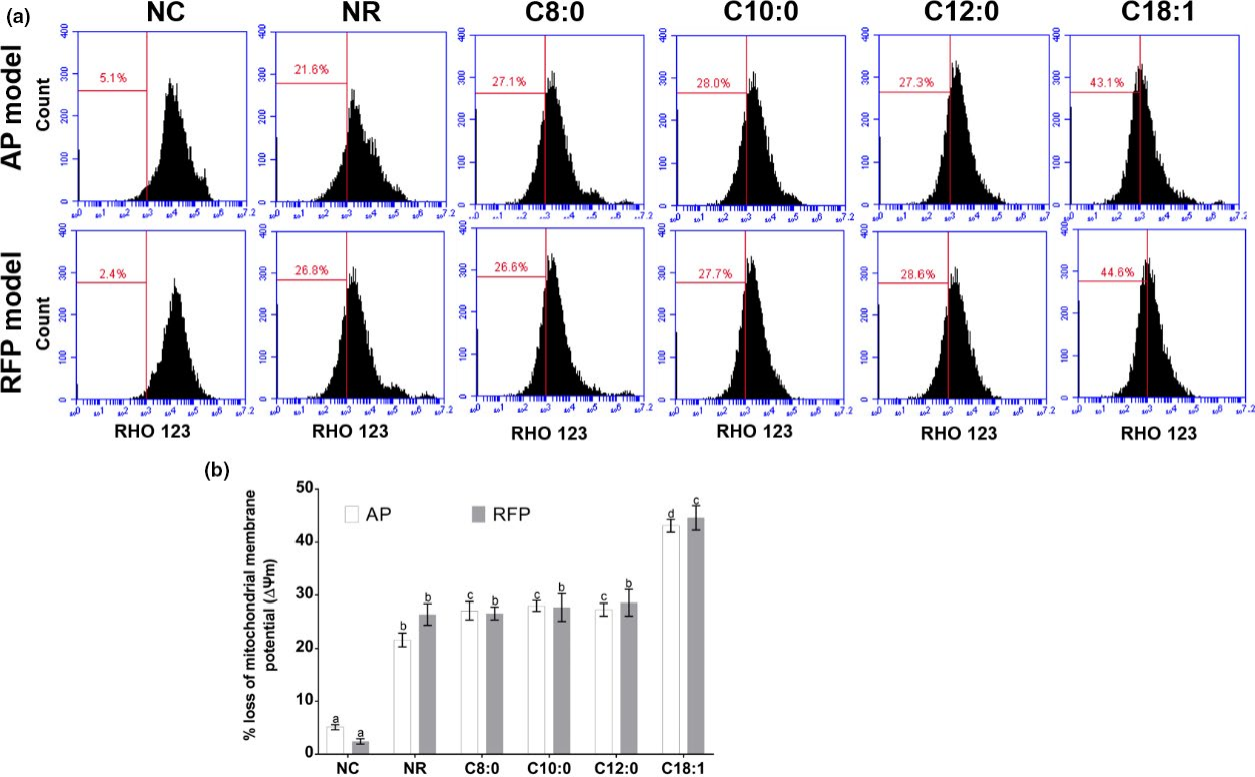
Effect of 200 μM FA on the mitochondrial membrane potential (ΔΨm) in AP‐ or RFP‐induced LO2 cell model. The cells treated with 200 μM FA for 24 hr were incubated with Rhodamine 123, and ΔΨm was measured by flow cytometry (a). The percentage loss of ΔΨm in the control and FA‐treated cells (b). Mean values (*n* = 3) with the same letter are not significantly different (*p* > .05)

Mitochondrial membrane potential (MMP) serves as a central regulator of cell health, which is associated with oxidative stress and apoptosis in the cell. In order to explore the effects of MCFA and LCFA on oxidative stress and apoptotic pathway in AP or RFP model, Rhodamine 123, a mitochondrion‐specific fluorescent dye, was used to determine the change in MMP by flow cytometry. As shown in Figure 4a,b, the fluorescence intensities were not significantly different between MCFA groups and NR group in RFP model. Compared with the LCFA group, the fluorescence intensity in MCFA groups increased from 66.9% to 72.9%, 72.0%, and 72.7% in AP model and from 65.4% to 73.4%, 72.3%, and 71.4% in RFP model, respectively. These results suggested that LCFA may decreased MMP, and MCFA did not affect MMP compared with the NR group in the AP or RFP model.

### 3.5 Effect of MCFA and LCFA on oxidative stress in AP‐ or RFP‐treated LO2 cells

Antioxidant capacity was examined by measuring activities of intracellular SOD and CAT, and the levels GSH, MDA, and T‐AOC in AP or RFP model following exposure to 200 µM MCFA and LCFA for 24 hr. As shown in Tables 2 and 3, the activities of SOD and CAT, and the levels of GSH, MDA, and T‐AOC in MCFA groups were similar to the NR group, whereas LCFA treatment significantly reduced the activities of SOD and CAT, and the levels of GSH and T‐AOC, and significantly increased the levels of MDA. These results indicated that treatment with MCFA did not produce the detrimental effects on oxidative stress, but when the cells were exposed to LCFA, oxidative stress may increase compared to the NR group in AP‐ or RFP‐treated LO2 cells.

**TABLE 2:** Effects of FA on oxidative stress in AP‐treated LO2 cells

Group	T‐AOC (U/mgprot)	MDA (nmol/mgprot)	SOD (U/mgprot)	GSH (µmol/gprot)	CAT (U/mgprot)
NC	0.956 ± 0.119^c^	1.060 ± 0.153^a^	20.110 ± 0.826^c^	8.838 ± 0.713^c^	1.810 ± 0.065^c^
NR	0.550 ± 0.074^b^	3.607 ± 0.664^b^	15.902 ± 1.014^b^	5.190 ± 0.486^b^	0.967 ± 0.061^b^
C8:0	0.535 ± 0.072^b^	3.529 ± 0.570^b^	16.402 ± 1.053^b^	5.399 ± 0.833^b^	1.024 ± 0.084^b^
C10:0	0.540 ± 0.096^b^	3.881 ± 0.860^b^	15.425 ± 0.702^b^	4.975 ± 0.886^b^	0.968 ± 0.137^b^
C12:0	0.513 ± 0.111^b^	4.086 ± 0.607^b^	15.074 ± 0.873^b^	4.703 ± 0.709^b^	0.959 ± 0.062^b^
C18:1	0.294 ± 0.062^a^	5.062 ± 0.677^c^	10.408 ± 1.362^a^	3.728 ± 0.496^a^	0.502 ± 0.077^a^

Note

Results are represented as the mean values ± standard deviations (*n* = 6). Different letters in the column represent significant differences at *p* < .05.

**TABLE 3:** Effects of FA on oxidative stress in RFP‐treated LO2 cells

Group	T‐AOC (U/mgprot)	MDA (nmol/mgprot)	SOD (U/mgprot)	GSH (µmol/gprot)	CAT (U/mgprot)
NC	1.156 ± 0.115^c^	1.328 ± 0.261^a^	21.549 ± 0.756^c^	9.194 ± 0.651^c^	1.961 ± 0.042^c^
NR	0.615 ± 0.088^b^	4.541 ± 0.748^b^	16.650 ± 0.964^b^	5.826 ± 1.051^b^	1.051 ± 0.051^b^
C8:0	0.586 ± 0.084^b^	4.197 ± 0.597^b^	17.049 ± 1.072^b^	5.845 ± 0.874^b^	0.988 ± 0.126^b^
C10:0	0.608 ± 0.099^b^	4.212 ± 0.509^b^	16.631 ± 0.910^b^	5.637 ± 1.056^b^	0.961 ± 0.130^b^
C12:0	0.525 ± 0.058^b^	4.781 ± 0.799^b^	15.756 ± 0.868^b^	5.325 ± 0.783^b^	0.904 ± 0.091^b^
C18:1	0.289 ± 0.084^a^	5.562 ± 0.658^c^	11.048 ± 1.216^a^	4.032 ± 0.926^a^	0.608 ± 0.106^a^

Note

Results are represented as the mean values ± standard deviations (*n* = 6). Different letters in the column represent significant differences at *p* < .05.

### 3.6 Effects of MCFA and LCFA on the expression of inflammatory cytokines in AP‐ or RFP‐treated LO2 cells

**FIGURE 5:**
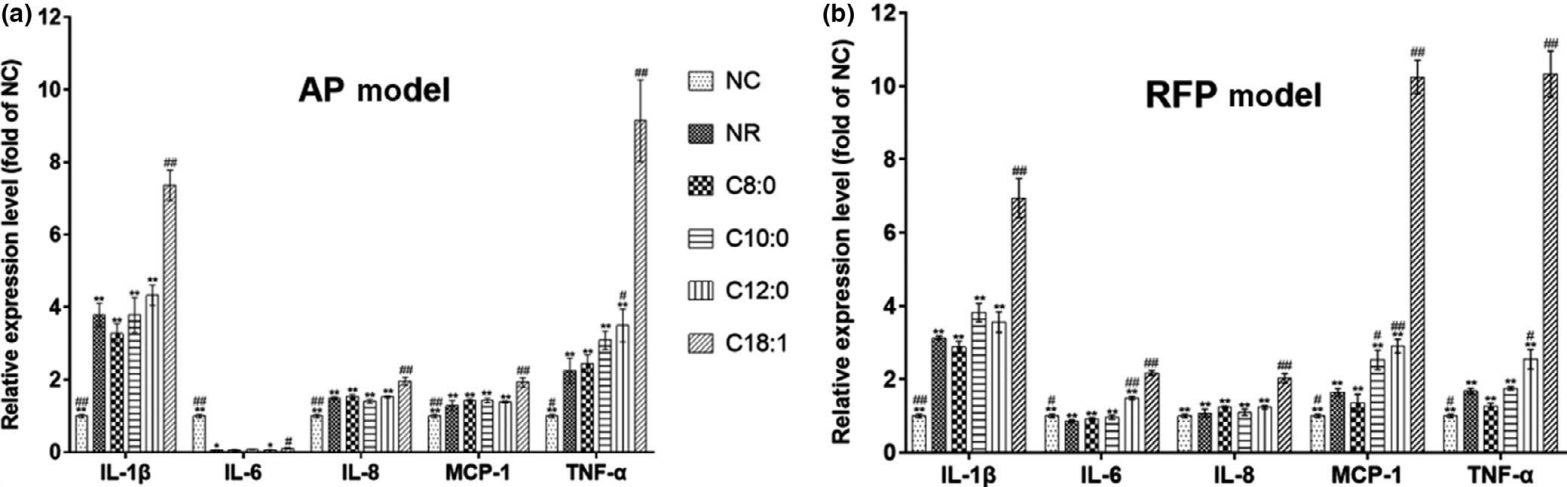
Effect of FA on mRNA expression levels of inflammatory cytokines in AP‐ and RFP‐induced LO2 cells as determined by q‐PCR. The mRNA expression levels of inflammatory cytokines in AP model (a) and RFP model (b). The relative expression ratio for each gene was presented as the ratio to the NC group. The housekeeping gene β‐actin was used as an internal control. Data are expressed as mean ± *SD* (*n* = 3). *p < .05, **p < .01 compared with the C18:1 group; ^#^ *p* < .05, ^##^ *p* < .01 compared with the NR group

As shown in Figure 5a,b, in the AP or RFP model, the mRNA expression levels of IL‐1β, IL‐6, IL‐8, MCP‐1, and TNF‐α were significantly increased in the C18:1 group compared with the NR group (*p* < .01). Additionally, in the AP model, the mRNA expression level of TNF‐α was significantly increased in the C12:0 group. Similarly, the expression levels of IL‐6, MCP‐1, and TNF‐α were markedly increased in the C12:0 group compared with the NR group (*p* < .05). Overall, the results showed that the mRNA expression levels of inflammatory cytokines in MCFA groups were significantly lower than the LCFA group.

**FIGURE 6:**
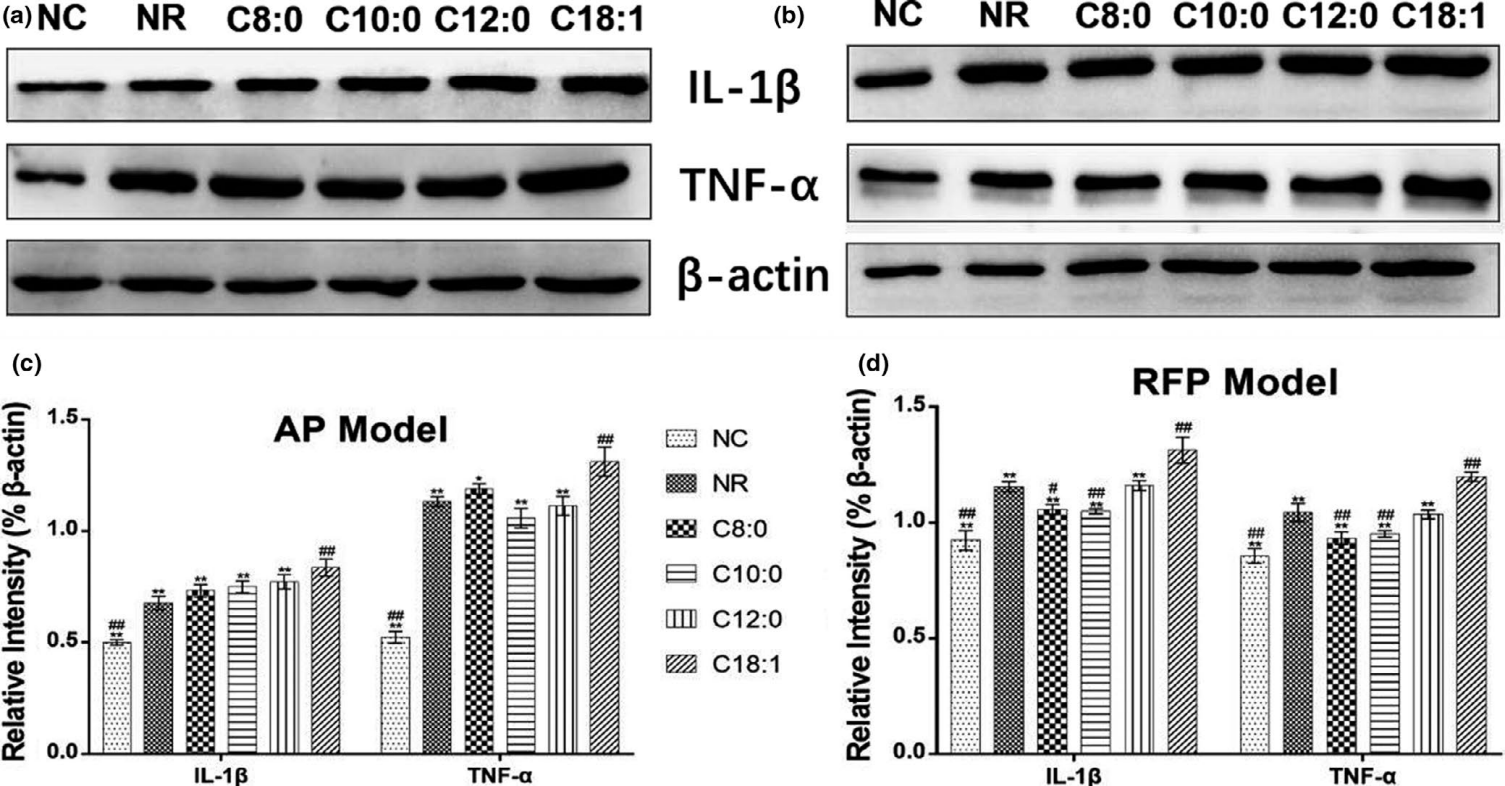
Protein expression of IL‐1β and TNF‐α in AP‐ and RFP‐induced LO2 cells treated with FA for 24 hr. Protein expression levels of IL‐1β and TNF‐α in AP model (a) and RFP model (b). Relative protein expression levels of IL‐1β and TNF‐α in AP model (c) and RFP model (d) shown as a percentage of β‐actin were presented. Data are expressed as mean ± *SD* (*n* = 3). *p < .05, **p < .01 compared with the C18:1 group; ^#^ *p* < .05, ^##^ *p* < .01 compared with the NR group

The effects of FA on protein expression of classical inflammatory cytokines (IL‐1β and TNF‐α) in AP or RFP model were further examined. As shown in Figure 6a,c, the levels of IL‐1β and TNF‐α were markedly reduced in the MCFA group compared with the LCFA group, and there were no significant differences between MCFA group and NR group in AP model. The expression levels of IL‐1β and TNF‐α were significantly decreased in both C8:0 and C10:0 groups compared with the NR group in the RFP model. Meanwhile, in contrast to LCFA treatment, the levels of IL‐1β and TNF‐α were substantially decreased by MCFA treatment. Taken together, these results indicated that, in comparison with the NR group, LCFA significantly up‐regulated the expression of inflammatory cytokines to increase the hepatotoxicity, but MCFA may not affect overall inflammation.

## 4 DISCUSSION

Drug‐induced liver injury, a potentially fatal adverse event, was often underestimated and underreported in epidemiological studies (Baskaran & Prince, 2017; Iorga, Dara, & Kaplowitz, 2017). It was reported that the annual incidence rates of DILI were 19.1 and 13.9 cases per 100,000 inhabitants in Iceland and France (Björnsson, Bergmann, Björnsson, Kvaran, & Sigurdur, 2013; Sgro et al., 2002). In recent years, over 1,000 drugs have been associated with DILI and the list is continuing to grow, which is also leading to the common abortion of new medicines approval and withdrawal of postmarketing drugs (Kaplowitz, Win, Than, Liu, & Dara, 2015; Senior, 2007; Stirnimann, Kessebohm, & Lauterburg, 2010; Teschke & Andrade, 2015). Both AP and RFP have been used to establish animal or cell liver injury model for screening antihepatotoxic and/or hepatoprotective activities of drugs (Akakpo et al., 2018; Darvin et al., 2018). As the concentration of AP or RFP increased and the treatment time was prolonged, the cell viability decreased in a dose‐ and time‐dependent manner (Figure 1a,b). The IC50 values for RFP were reported to be 548.91 and 234.96 µM in 24 and 48 hr, respectively, in LO2 cells (Zhang & Xu, 2018). Other studies found that the IC50 value for RFP in 24 hr was 530.33 µM and established cell injury model by treatment with 10 mM AP for 24 hr (Wu et al., 2016; Yuan‐Jing, Wei, Jian‐Ping, Yu‐Xia, & Zi‐Ling, 2016). These reports were similar to our results in this study. Our results showed that the optimal damage condition of the DILI cell model was treatment with 10 mM AP or 600 µM RFP in LO2 cells for 24 hr, and the treated cells had low cell viability, an increased cell apoptosis rate, and elevated leakage levels of ALT, AST, and LDH (Figure 1). LO2 cells treated with AP or RFP also exhibited increased oxidative stress and lipid peroxidation as assessed by MDA production (Tables 2 and 3).

Medium‐chain fatty acids are absorbed easily as they are transported to the liver directly, which are not incorporated into chylomicrons. And they can supply energy rapidly because they could readily penetrate the mitochondrial membrane and do not need a carnitine shuttle system and carnitine palmitoyltransferase (Zhou, Wang, Jiang, & Yu, 2017). In contrast, LCFA are slowly absorbed, because they must be incorporated into chylomicrons and rely on the lymphatic and vascular system to transport (Poppitt et al., 2010). Therefore, LCFA are preferentially stored as body fat even liver fat and may easily induce hepatocyte injury as they have different pharmacokinetics and utilize different metabolic pathways compared with MCFA (Kim, Choe, et al., 2017; Kim, Nam, et al., 2017; Lemarié, Beauchamp, Legrand, & Rioux, 2016). In addition, a diet containing high MCFA could significantly reduce body fat accumulation, lower insulin resistance, as well as ameliorate apoptosis, oxidative stress, and inflammatory responses as compared to LCFA diet in rats (Fu, Zeng, Zeng, Wang, & Gong, 2016; Fu, Zeng, Zeng, Wang, Wen, et al., 2016; Hu, Shen, Xiong, Zhu, & Deng, 2018; Zhou, Wang, Jiang, Zhang, et al., 2017). These factors (apoptosis, oxidative stress, and inflammatory responses) may be the causes of DILI. Thus, the effects of MCFA on the hepatotoxicity induced by drugs in human liver cells compared with LCFA (oleic acid) are worth to be studied.

It has been reported that treatment with less than 200 µM FA (octanoic acid, decanoic acid, lauric acid, and oleic acid) did not cause obvious toxicity in LO2 cells (Li et al., 2016). It was found that MCFA did not cause apoptosis, oxidative stress, and inflammation in the hepatic cells with steatosis compared with LCFA (Wang et al., 2016). Data from the present study (Figure 2) have shown that the proliferation rates of LO2 cells were reduced when the concentrations of FA were higher than 200 µM and treatment with 200 µM MCFA did not aggravate cell injury compared with treatment with LCFA in AP or RFP model. Previous studies reported that the ingestion of overdose AP could cause overproduction of reactive metabolite N‐acetyl‐p‐benzoquinoneimine (NAPQI) by cytochrome P450 2E1 isozyme. The overproduction of NAPQI led to GSH depletion and presumably altered protein functions by covalently binding to cellular or mitochondrial proteins, eventually leading to mitochondrial dysfunction, oxidative stress, and even hepatocyte necrosis (Huo et al., 2017; Jaeschke & Ramachandran, 2018; Lv et al., 2018; Zhang et al., 2017). Meantime, recent studies suggested that endoplasmic reticulum stress and cytochrome P450‐mediated pathways may play important roles in RFP‐induced hepatocellular damage, in addition to oxidative stress, apoptosis, mitochondrial dysfunction, and cholestasis (Benson et al., 2016; Kim, Choe, et al., 2017; Kim, Nam, et al., 2017). It was reported that AP‐induced LO2 cells injury may be dominated by necrosis or programmed necrosis and may not involve relevant apoptosis because the fundamental difference between primary hepatocytes and hepatoma cells is the dramatically lower expression of cytochrome P450 enzymes, which are responsible for the toxicity (Jaeschke, Duan, Akakpo, Farhood, & Ramachandran, 2018). Oxidative stress, inflammation, and cell apoptosis were the extensively accepted causes for AP‐ or RFP‐induced liver injury. Hence, we investigated the effects of medium‐ and long‐chain fatty acids on AP‐ or RFP‐induced hepatocellular injury by mitochondrial dysfunction, oxidative stress, inflammatory response, and apoptotic pathways. Our data confirmed that the administration of LCFA to AP or RFP model deteriorated oxidative stress, as shown by the elevated activities of SOD and CAT and levels of T‐AOC and GSH as well as a reduced level of MDA compared to the NR group (Tables 2 and 3). Additionally, the administration of LCFA to AP or RFP model significantly aggravated mitochondrial dysfunction concurrent with increased apoptosis rate as well as enhanced expression of inflammatory cytokines compared to the NR group (Figures 4, 5, 6), which supported reduced cell proliferation and the increased leakage of ALT, AST, and LDH. However, our results also showed that the apoptosis rate and the parameters of oxidative stress and inflammation remained unchanged in the MCFA groups compared with the NR group in AP or RFP model. Therefore, we proposed that MCFA may be safer than LCFA as nutrition support or the selection of daily oil and fat for the patients with DILI. Surely, to further confirm whether the effects of MCFA were mediated by oxidative stress, inflammatory response, and apoptotic pathways, we need to determine the expression of related signaling pathways such as sirtuin 1 (SIRT1), c‐Jun N‐terminal kinase (JNK), and nuclear factor erythroid 2‐related factor 2/Kelch‐like ech‐associated protein 1 (Nrf2/Keap1) pathways in subsequent experiments (Akakpo et al., 2018; Rada et al., 2017; Yuan‐Jing et al., 2016).

In conclusion, our data have shown that treatment with 10 mM AP or 600 µM RFP for 24 hr was the optimum condition for the establishment of the DILI model in LO2 cells. Our results demonstrated that treatment with LCFA may deteriorate DILI by reducing activities of antioxidant enzymes, enhancing lipid peroxidation, inducing apoptosis, and up‐regulating inflammatory cytokine expression. On the contrary, treatment with MCFA did not produce the detrimental effects and may attenuate inflammation or slightly improve oxidative stress on DILI. Therefore, this work revealed the effects of different FA on DILI and also meant it is important to consider the effect of FA on human health in further studies.

## CONFLICT OF INTEREST

The authors declare no financial or commercial conflict of interest.

## ETHICAL APPROVAL

This study does not involve any human or animal testing.

## Data Availability

The data that support the findings of this study are available from the corresponding author upon reasonable request.
